# RETRACTION: Apo A1 Mimetic Rescues the Diabetic Phenotype of HO‐2 Knockout Mice via an Increase in HO‐1 Adiponectin and LKBI Signaling Pathway

**DOI:** 10.1155/ijhy/9782561

**Published:** 2026-03-25

**Authors:** International Journal of Hypertension

RETRACTION: J. Cao, N. Puri, K. Sodhi, L. Bellner, N. G. Abraham, and A. Kappas, “Apo A1 Mimetic Rescues the Diabetic Phenotype of HO‐2 Knockout Mice via an Increase in HO‐1 Adiponectin and LKBI Signaling Pathway,” *International Journal of Hypertension*, 2012, 628147, https://doi.org/10.1155/2012/628147.

The above article, published online on 04 April 2012 in Wiley Online Library (https://wileyonlinelibrary.com), has been retracted by agreement between the journal Editor‐in‐Chief, Franco Veglio; and John Wiley & Sons Ltd. The retraction has been agreed due to concerns identified with the figures, both directly and on PubPeer [1].

Specifically:

• The Actin bands shown in Figures 3b and 4a appear identical to the Actin bands shown in Figure 6a of [2].
• The Actin bands shown in Figures 3b and 4a appear to be 180‐degree vertically flipped versions of the Actin bands shown in Figures 6c and 6d of [2].
• The ß‐Actin bands shown in Figure 4c appear to be 180‐degree horizontally flipped versions of the Actin bands shown in Figures 6c and 6d of [2].

Following an assessment of the concerns raised, together with the author’s inability to provide the raw data and original images, the Editorial Board has concluded that the results and conclusions can no longer be considered reliable. The article is therefore retracted.

The authors did not respond to the retraction.
